# Blunt rupture of the right hemidiaphragm with herniation of the right colon and right lobe of the liver

**DOI:** 10.4103/0974-2700.58659

**Published:** 2010

**Authors:** Anjana Bairagi, Saundrarajen R Moodley, Timothy C Hardcastle, David J Muckart

**Affiliations:** Department of Surgery, University of KwaZulu Natal, Mayville, 4058, South Africa; 1 Trauma Intensive Care Unit, Inkosi Albert Luthuli Central Hospital, Private Bag X03, Mayville, 4058, South Africa

**Keywords:** Blunt, diaphragm rupture, herniation, right sided

## Abstract

Acute right hemidiaphragm rupture with abdominal visceral herniation is reportedly less common than on the left. We present a complex case of blunt rupture of the right hemidiaphragm with herniation of the right colon and right lobe of the liver in a multiply injured patient. The diagnostic approach, with specific reference to the imaging studies, and surgical management is discussed, followed by a brief literature review highlighting the complexities of the case.

## INTRODUCTION

Traumatic diaphragm rupture (TDR) was first described by Sennertus in 1541.[1,2] In 1886, Riolfi was credited with the first successful repair of a diaphragmatic laceration.[1,2] Following blunt thoracoabdominal trauma, diaphragmatic rupture is reported in 0.8–3.6% of patients.[3] Right-sided diaphragmatic rupture is rare and occurs in approximately 5–20% of all diaphragmatic disruptions.[4] Herniation of intra-abdominal organs into the pleural cavity is uncommon, with an incidence of only 19% with right-sided TDR.[4] The diagnosis is often difficult in a polytrauma patient and a delay in diagnosis is implicated in increased mortality and morbidity. This report details a right-sided TDR with herniation of the right colon and right lobe of the liver into the right chest cavity.

## CASE REPORT

**Figure 1:**
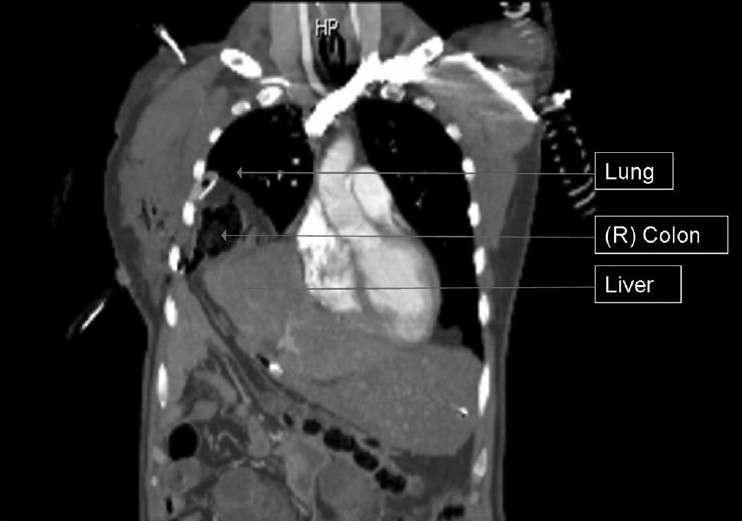
Coronal computed tomography scan demonstrating hernia of the right diaphragm with right colon and right liver lobe in the right chest cavity

**Figure 2:**
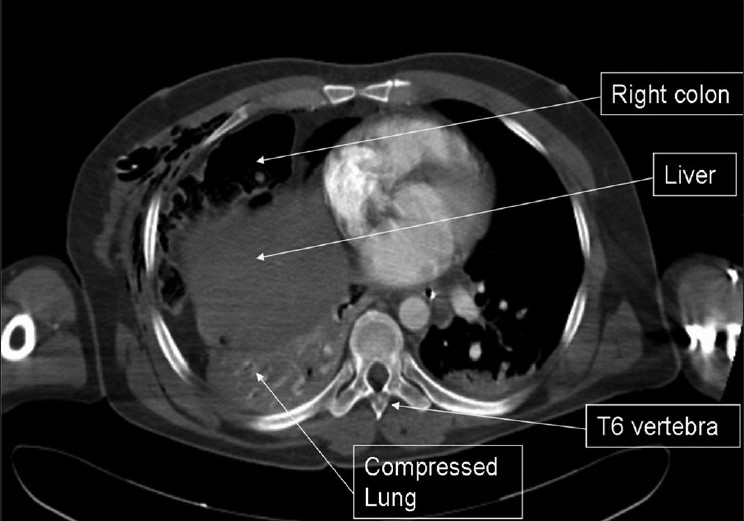
Axial computed tomography scan demonstrating right colon and right liver lobe in the right chest cavity with compression of the lung parenchyma

A 40-year-old male involved in a motor vehicle collision as an unrestrained occupant sustained multiple injuries after being ejected from the vehicle. He was intubated and ventilated on the scene. The initial blood pressure was 105/55 and pulse was 102. His oxygen saturation was 98% on 50% oxygen with Synchronized Intermittent Mandatory Ventilation-pressure-limited ventilation. He was referred to this major trauma center with the following injuries: closed head injury, right hemothorax, bilateral pulmonary contusions, more extensive on the right, multiple fractures of right-sided ribs and vertebrae (C7, L1-3) and pelvic fracture with frank hematuria. A chest tube (ICD) was placed in the trauma bay for the hemothorax identified on clinical examination. Standard fluid resuscitation was initiated. Additionally, a chest X-ray revealed an elevated right hemidiaphragm. Because he was hemodynamically stable after initial assessment and resuscitation, he underwent a trauma computed tomography angiogram (CTA) [Figures 1 and 2], which revealed liver and right colon herniating into the right pleural space and a small amount of intra-abdominal free fluid. Exploratory laparotomy confirmed a large posterior ruptured right hemidiaphragm extending to the right half of the central tendon with a 5 mm rim of diaphragm posterior and exposed pericardium. The hepatic flexure with right lobe of the liver had herniated into the thoracic cavity and two segments of devitalized small bowel were found in the abdomen. The bowel and liver were reduced into the abdominal cavity. A single layer repair of the diaphragm with non-absorbable suture was performed, the small bowel resected and primary anastamoses were performed. He experienced a complicated post-operative course. A tracheostomy was inserted for long-term airway and ventilation management. He was also treated for catheter-related blood stream infection and intensive care unit delirium. He was discharged to a step-down hospital with a view to further rehabilitation.

## DISCUSSION

The weakest point of the diaphragm is the left posterolateral area along the embryonic fusion lines of the pleuroperitoneal membrane. This is the most common site for diaphragm rupture. The accepted mechanisms of rupture include a sudden increase in intra-abdominal pressure throughout the abdomen with the relatively weak, unprotected left diaphragm tearing from that force, or avulsion of the attachments of the diaphragm or shearing of the stretched membrane after right or left lateral impact, the chest wall and rib fracture fragments directly penetrating the diaphragm wall. It has also been demonstrated experimentally that the right hemidiaphragm is mechanically stronger than the left and thus requires a larger force for disruption. The right hemidiaphragm is partially protected from abdominal impact by the energy-absorbing liver.[5,6] The pre-operative diagnosis of TDR is difficult.[3,5,6] Blunt rupture of the right hemidiaphragm with liver hernia into the chest is a rare injury, which may be initially overlooked in a polytrauma patient. In this case, the patient sustained blunt trauma to both thorax and abdomen, with pelvic fractures arousing a high index of suspicion for the diagnosis. Diagnostic modalities implemented in assessing TDR include chest X-ray, upper intestinal tract contrast studies, sonography, CT scan, magnetic resonance imaging (MRI) and laparoscopy. It is important that the diagnostic methods utilized are dependent on the stability of the patient, the facilities available at the time of presentation to hospital and operator skill. Chest radiographs have a relatively low sensitivity for diagnosis of TDR, but remain a screening tool with findings suggestive of the diagnosis in only 17–40% of patients.[4] In this patient, an elevated right hemidiaphragm was identified on X-ray. CT scan is the diagnostic modality of choice to differentiate the causes of an elevated right hemidiaphragm and facilitate diagnosis. The limitations in diagnosis with axial CT scans have been improved upon with the advent of helical CT scans. The main diagnostic signs of diaphragm rupture on CT scan are direct visualization of diaphragm discontinuity, intrathoracic visceral or omental fat herniation, the “collar sign,” which is a waist-like constriction of the herniating viscera and the “dependent viscera sign,” which is a direct contact of the herniated liver with the chest wall without intervening lung and thickening of the hemidiaphragm from hemorrhage.[5,7] In this patient, the following signs were found on CTA: “dependent viscera sign” and thickening of the right hemidiaphragm due to hemorrhage. The “collar sign” was not seen as the diaphragm laceration was so large that it did not cause identifiable constriction of the herniated liver and bowel on CTA. Helical CT has become the preferred imaging modality due to its ability to acquire volumetric data and good-quality coronal and sagittal reformatted images.[5,8] The published diagnostic CT sensitivity and specificity of right hemidiaphragm rupture is 50–90% and 90–100%, respectively.[3,4,9] MRI offers identical information to that of helical CT but with direct coronal and sagittal images and better spatial and contrast resolutions of anatomic structures.[9] However, MRI has limitations, including restricted patient access, cost and, lastly, MRI is currently unsuitable for acute emergency patients. Current literature recommends that the immediate surgical management of TDR should be via an abdominal approach. This facilitates identification of associated intra-abdominal injuries, found in 30–70% of patients.[3] Additionally, herniations can be easily reduced to the abdomen and, although more challenging, the defect in the diaphragm can be approximated primarily in one or two layers. This often negates the need for synthetic grafts. The mortality associated with right TDR within the first 24 h is reported to be 20–31%.[4,8] In this patient, an abdominal approach was utilized. This enabled identification of the devitalized small bowel not visible on CT scan. Closure of the diaphragm was technically challenging as approximation of the edges of the large rent was made difficult by the miniscule 5-mm posterior rim of the diaphragm. Thoracotomy is recommended for patients with delayed presentation, concomitant thoracic injuries and large hernias or an associated empyema.

## CONCLUSION

In conclusion, diagnosis of right-sided blunt diaphragmatic rupture requires a high index of suspicion. Although, chest X-ray is the most common imaging tool, CTA is the preferred imaging modality. Immediate surgical repair via an abdominal approach to repair the diaphragm and reduce herniated viscera constitutes the recommended acute surgical management in the severely injured patient. Survival largely depends on the presence of associated injuries.[6]
